# Multitarget Stool DNA Screening in Clinical Practice: High Positive Predictive Value for Colorectal Neoplasia Regardless of Exposure to Previous Colonoscopy

**DOI:** 10.14309/ajg.0000000000000546

**Published:** 2020-02-17

**Authors:** Jason D. Eckmann, Derek W. Ebner, Jamie Bering, Allon Kahn, Eduardo Rodriguez, Mary E. Devens, Kari L. Lowrie, Karen Doering, Sara Then, Kelli N. Burger, Douglas W. Mahoney, David O. Prichard, Michael B. Wallace, Suryakanth R. Gurudu, Lila J. Finney, Paul Limburg, Barry Berger, David A. Ahlquist, John B. Kisiel

**Affiliations:** 1 Department of Internal Medicine, Mayo Clinic, Rochester, Minnesota, USA;; 2 Department of Internal Medicine, Mayo Clinic, Scottsdale, Arizona, USA;; 3 Division of Gastroenterology and Hepatology, Mayo Clinic, Scottsdale, Arizona, USA;; 4 Division of Gastroenterology and Hepatology, Mayo Clinic, Rochester, Minnesota, USA;; 5 Division of Biomedical Statistics and Informatics, Mayo Clinic, Rochester, Minnesota, USA;; 6 Division of Gastroenterology and Hepatology, Mayo Clinic Health System, LaCrosse, Wisconsin, USA;; 7 Division of Gastroenterology and Hepatology, Mayo Clinic, Jacksonville, Florida, USA;; 8 Division of Health Care Policy and Research, Mayo Clinic, Rochester, Minnesota, USA;; 9 Exact Sciences, Madison, Wisconsin, USA.

## Abstract

**OBJECTIVES:**

Multitarget stool DNA (MT-sDNA) testing has grown as a noninvasive screening modality for colorectal cancer (CRC), but real-world clinical data are limited in the post-FDA approval setting. The effect of previous colonoscopy on MT-sDNA performance is not known. We aimed to evaluate findings of colorectal neoplasia (CRN) at diagnostic colonoscopy in patients with positive MT-sDNA testing, stratified by patient exposure to previous colonoscopy.

**METHODS:**

We identified consecutive patients completing MT-sDNA testing over a 39-month period and reviewed the records of those with positive tests for neoplastic findings at diagnostic colonoscopy. MT-sDNA test positivity rate, adherence to diagnostic colonoscopy, and the positive predictive value (PPV) of MT-sDNA for any CRN and neoplastic subtypes were calculated.

**RESULTS:**

Of 16,469 MT-sDNA tests completed, testing returned positive in 2,326 (14.1%) patients. After exclusion of patients at increased risk for CRC, 1,801 patients remained, 1,558 (87%) of whom underwent diagnostic colonoscopy; 918 of 1,558 (59%) of these patients had undergone previous colonoscopy, whereas 640 (41%) had not. Any CRN was found in 1,046 of 1,558 patients (PPV = 67%). More neoplastic lesions were found in patients without previous colonoscopy (73%); however, the rates remained high among those who had undergone previous colonoscopy (63%, *P* < 0.0001). The large majority (79%) of patients had right-sided neoplasia.

**DISCUSSION:**

MT-sDNA has a high PPV for any CRN regardless of exposure to previous colonoscopy. Right-sided CRN was found at colonoscopy in most patients with positive MT-sDNA testing, representing a potential advantage over other currently available screening modalities for CRC.

## INTRODUCTION

Colorectal cancer (CRC) is the second leading cause of cancer-related death in the United States (US) (1). Based on Surveillance, Epidemiology, and End Results (SEER) Program estimates, in 2019 alone, there will be over 145,000 new CRC diagnoses, with approximately 51,000 deaths attributed to CRC (2). Recent national trends demonstrate an overall decline in CRC mortality rates (2). This appears to be due in large part to the introduction of successful screening programs which have led to increased detection of precancerous colorectal neoplasia (CRN) and early stage CRC (3–5). However, approximately 1 in 3 screen-eligible adults has not participated in CRC screening, leaving over 23 million US adults without these benefits (6,7).

Colonoscopy, the most widely used screening modality in the United States, has been shown in observational studies to reduce mortality from CRC (6,8,9) but has important limitations. First, protective effects are reduced in the right colon compared with the left, believed to be largely because of missed lesions proximal to the splenic flexure (9–15). Second, there is significant interoperator inconsistency in colonoscopy quality as shown by wide variations in adenoma detection rates (16–19), which are particularly pronounced in the right colon (20). Operator variability is clinically important because adenoma detection rates have been shown to be inversely correlated with the development of “interval CRCs,” defined as cancers diagnosed within 10 years of a “negative” screening colonoscopy for average-risk patients (16,21,22). Finally, the invasiveness of colonoscopy and the potential lost time from work related to the procedure are barriers for many patients when considering screening options (23).

Among noninvasive screening tests, guaiac-based fecal occult blood testing (FOBT) has been shown in randomized controlled trials to reduce CRC-related mortality; however, the benefit appears to be primarily for left-sided cancers (24,25). This bias is also observed for the more sensitive fecal immunochemical test (FIT) (25,26). Furthermore, both FIT and FOBT assays have poor sensitivity for sessile serrated polyps (SSPs) (27–30), which are typically nonhemorrhagic, occur predominantly in the proximal colon, and are estimated to cause up to one-third of all CRC (31–34). Finally, adherence to the recommended annual screening for these modalities remains suboptimal (35–37), with studies finding that only 14% and <1% of patients completed annual screening over 5 and 10 year periods, respectively (38,39).

Multitarget stool DNA (MT-sDNA) testing was developed as an alternative noninvasive method of CRC screening. The MT-sDNA assay amplifies methylated *BMP3* and *NDRG4*, mutant *KRAS*, and *β-actin* and measures hemoglobin through immunochemical testing (40). The test was approved in 2014 by the US Food and Drug Administration (FDA) after demonstration of high sensitivity for both left- and right-sided CRCs and high-risk precancers in a large cross-sectional screen-setting study (41). These findings were replicated in a second screen-setting study of Alaska Natives (42). Furthermore, MT-sDNA has subsequently been shown to have superior sensitivity for detecting SSPs compared with other noninvasive fecal blood tests (43). MT-sDNA utilization has increased substantially since its approval and subsequent inclusion in the US Preventive Services Task Force CRC screening guidelines (5); promising postapproval data on test performance are now emerging on a limited scale (44,45).

In the present study, at a large multisetting practice using MT-sDNA for CRC screening, we aimed to measure the overall MT-sDNA test positivity rate, assess yield of colorectal neoplastic lesions in test-positive cases (positive predictive value [PPV]), and describe adherence to subsequent diagnostic colonoscopy among MT-sDNA-positive patients. We hypothesized that previous exposure to screening colonoscopy may alter lesion prevalence and, therefore, affect test PPV for CRN. Accordingly, we sought to evaluate the PPV of MT-sDNA for all CRN and by lesion subtype based on site, histology, and the presence/absence of advanced features. These features were evaluated both in the overall cohort as well as in groups stratified by patients' previous exposure to screening colonoscopy. These metrics will provide real-world performance characteristics of MT-sDNA as a screening modality for CRC in clinical practice, which is critical to informing both patient and provider expectations after positive MT-sDNA testing.

## METHODS

### Study overview

This was a retrospective consecutive series cohort study which included all patients with MT-sDNA tests (Cologuard; Exact Sciences, Madison, WI) completed at any Mayo Clinic site between October 1, 2014, and December 31, 2017. Mayo Clinic sites included tertiary referral centers (Rochester, MN; Scottsdale & Phoenix, Arizona; and Jacksonville, FL) and community practices within the Mayo Clinic Health System (Western Wisconsin, Southern Minnesota and Northern Iowa). The study was approved by the Mayo Clinic Institutional Review Board.

### Patient population

All patients aged 50 years and older undergoing MT-sDNA testing over the study period, and the subset of those with positive test results, were identified through a data pull performed by the Department of Biomedical Statistics and Informatics using diagnostic and procedure billing codes. To ensure accuracy of these data, this list was validated against data from the test manufacturer and a separate organizational laboratory data repository. Patients receiving care in Minnesota were required to have signed authorization for research-related medical record review in accordance with the Minnesota state law. The electronic medical record for each MT-sDNA test-positive patient was then reviewed, with data compiled in a secure centralized database (REDCap; Vanderbilt University, Nashville, TN). A midpoint review to ensure accuracy of data abstraction was performed by a single examiner (J.D.E.).

Patients were excluded if they were found to have any of the following risk factors for CRC: inflammatory bowel disease, known genetic syndrome predisposing to CRC, a history of aerodigestive tract malignancy, or a first-degree relative with CRC diagnosed at 60 years of age or younger. Patients with signs or symptoms of bleeding (+FOBT [<6 months], overt rectal bleeding [<3 months], or iron deficiency anemia [<3 months]), or a personal history of advanced CRN were also excluded. Patients with 3 or more polyps <1 cm in size were included among those with nonadvanced adenomas, in keeping with the FDA labeling for MT-sDNA.

### Data abstraction

For all average-risk MT-sDNA-positive patients, the date and site of MT-sDNA testing, as well as baseline demographic information including age at the time of MT-sDNA testing, sex, race, and tobacco use, were extracted through electronic data pulls. From diagnostic colonoscopy reports, we abstracted the date of diagnostic colonoscopy, neoplastic and non-neoplastic findings, withdrawal time, and photo documentation of cecal intubation. All patients were included in the primary analysis regardless of the quality of colonoscopy preparation. If the initial diagnostic colonoscopy was inadequate, only the subsequent adequate colonoscopy was included in the analysis. Information was also compiled regarding exposure to previous screening colonoscopy, including date and neoplastic findings.

Neoplastic endpoints at diagnostic colonoscopy included any CRN, advanced CRN, right-sided CRN, SSPs, and CRC. All neoplastic lesions were confirmed by clinical histopathology reports; no discarded or unretrieved lesions were included. Advanced CRN was defined as CRC or adenomas or SSPs with at least one of the following characteristics: ≥1 cm in size, high grade dysplasia, or having ≥25% villous elements. The lesion size was obtained from pathology reports for polyps removed *en bloc* and from endoscopic estimates for lesions removed in a piecemeal fashion. The lesion location was also recorded, with right-sided CRN defined as any polyp located at or proximal to the splenic flexure, and left-sided CRN located distal to the splenic flexure. For patients who underwent diagnostic colonoscopy at an outside institution (n = 121), primary source documents were used for data abstraction when available.

### Statistical analysis

Primary study endpoints including the rate of test positivity and adherence with diagnostic colonoscopy were described quantitatively. The PPV of MT-sDNA was calculated for any CRN and for each CRN subcategory. The proportional contribution of each subcategory to total CRN was also measured. These findings were then stratified by exposure to previous screening colonoscopy. No *a priori* power calculation was performed as this study summarizes a consecutive series patient population. Continuous variables were reported as medians and interquartile range (IQR) and compared using the Wilcoxon rank-sum test. Discrete variables were represented as percentages, with comparisons between subgroups using the Fisher exact test or χ^2^ test, where appropriate.

## RESULTS

### Study population and test positivity rate

**Figure 1.**
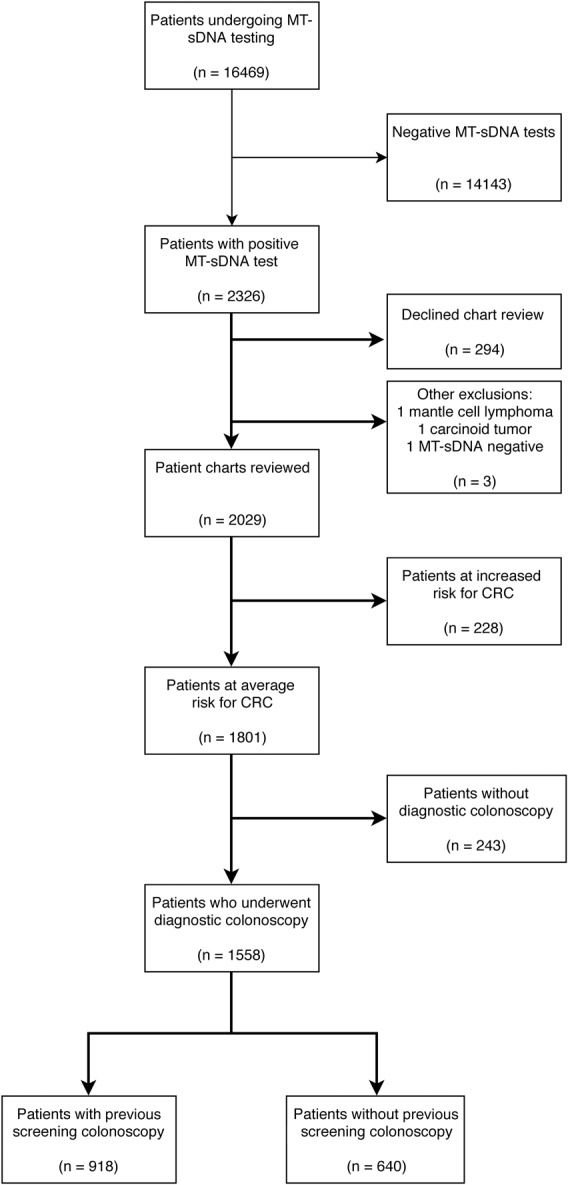
Study flow diagram. CRC, colorectal cancer; MT-sDNA, multitarget stool DNA.

A total of 16,469 tests were completed over the study time period, 2,326 of which were positive, resulting in an overall test-positive rate of 14.1% (Figure 1). Two external deidentified data sources were used to corroborate this estimate. Compared with the 2,326 positive tests we identified using internal billing data for our analysis, our figure was not more than ± 3 patients (0.1%) different than the other data sources. Of these 2,326 patients, research-related medical record review was not authorized by 294 individuals who were consequently excluded. Three additional patients were excluded; 2 with other gastrointestinal malignancies (mantle cell lymphoma and carcinoid tumor) and one who was found to have a negative MT-sDNA testing result that was discordant with the billing data.

Of the remaining 2,029 patients with positive MT-sDNA test results, 228 (11%) patients were at increased risk for CRC and were excluded from analysis (Supplemental Table 1, http://links.lww.com/AJG/B401). Of the 1,801 average-risk patients, 1,558 (87%) patients underwent diagnostic colonoscopy at a median of 44 (IQR 28–72) days after positive MT-sDNA test. Thirteen percent (243) of the patients had not obtained diagnostic colonoscopy by the time of database closure on April 1st, 2018. Of those undergoing diagnostic colonoscopy, 918 of 1,558 (59%) had undergone previous screening colonoscopy and 640 (41%) had not. Findings at previous colonoscopy did not place these patients at increased risk for CRC and are reported in Supplemental Table 2, http://links.lww.com/AJG/B402. The median age of patients with previous colonoscopy (70 [IQR 65–76] years) was significantly higher than those without previous screening (63 [IQR 55–69] years, *P* < 0.0001). Of the patients who had not undergone previous screening colonoscopy, 381 of 640 (60%) were older than 60 years. At each of the 3 major referral sites, most patients lived within a 100 mile radius: 680 of 897 (76%) in Rochester, 134 of 172 (78%) in Scottsdale, and 103 of 118 (87%) in Jacksonville. Other baseline characteristics are shown in Table 1.

**Table 1.**
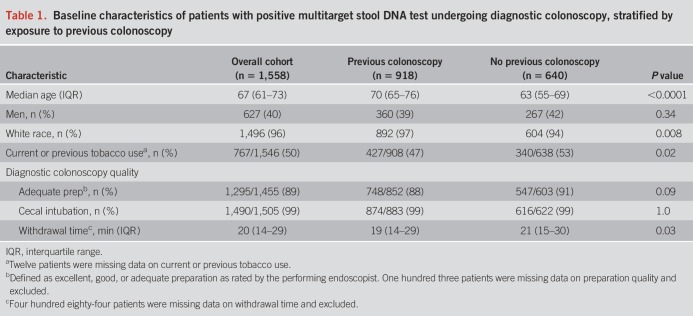
Baseline characteristics of patients with positive multitarget stool DNA test undergoing diagnostic colonoscopy, stratified by exposure to previous colonoscopy

### Positive predictive value

**Figure 2.**
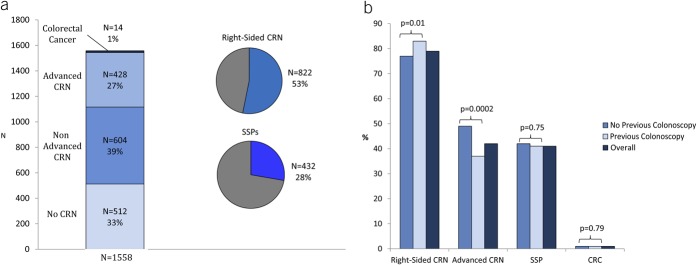
(**a**) Yield of neoplastic findings at diagnostic colonoscopy for all patients with MT-sDNA-positive tests. (**b**) Proportion of neoplastic lesions among patients with colorectal neoplasia at diagnostic colonoscopy after positive MT-sDNA test, stratified by exposure to previous screening colonoscopy. CRN, colorectal neoplasia; MT-sDNA, multitarget stool DNA.

Among the 1,558 MT-sDNA test-positive patients who underwent subsequent diagnostic colonoscopy, neoplastic lesions were discovered in 1,046, resulting in a PPV of 67% (95% confidence interval (CI) [65,69]) for any CRN. Advanced CRN was found in 442 patients (PPV 28%, 95% CI [26, 31]), and SSPs of any size were found in 432 patients (PPV 28%, 95% CI [26, 31]) (Figure 2A). Any CRN was more commonly detected at diagnostic colonoscopies performed at Mayo Clinic than the 121 examinations performed at outside institutions (PPV 68% vs 58%, *P* = 0.03). There was no significant difference in the PPV for advanced CRN at diagnostic colonoscopies performed at Mayo Clinic vs outside institutions (29% vs 26%, *P* = 0.67).

Of those with neoplasia, 42% had advanced lesions and 41% harbored SSPs (Figure 2B). Right-sided CRN was documented in 822 (79%, 95% CI [77, 82]) of the 1,034 patients in whom polyp location was documented, with an overall PPV for right-sided CRN of 53% (95% CI [51, 56]). Twelve patients did not have a polyp location recorded and were not included in this calculation. A median of 2 (IQR 0–4) polyps per patient were found in the overall cohort; 28 patients did not have accurate documentation of polyp number on their endoscopic report and were not included in this analysis.

Fourteen (1%) of the 1,558 patients with positive MT-sDNA tests undergoing diagnostic colonoscopy were found to have CRC. The median age at MT-sDNA testing which led to diagnosis of CRC was 66 (IQR 60–73) years. Eleven patients (79%) were found to have AJCC stage 0–II disease, whereas 3 (21%) harbored locally advanced or metastatic CRC (AJCC stage III–IV) (Table 2). CRCs were right-sided in 9 of 14 (64%) patients.

**Table 2.**
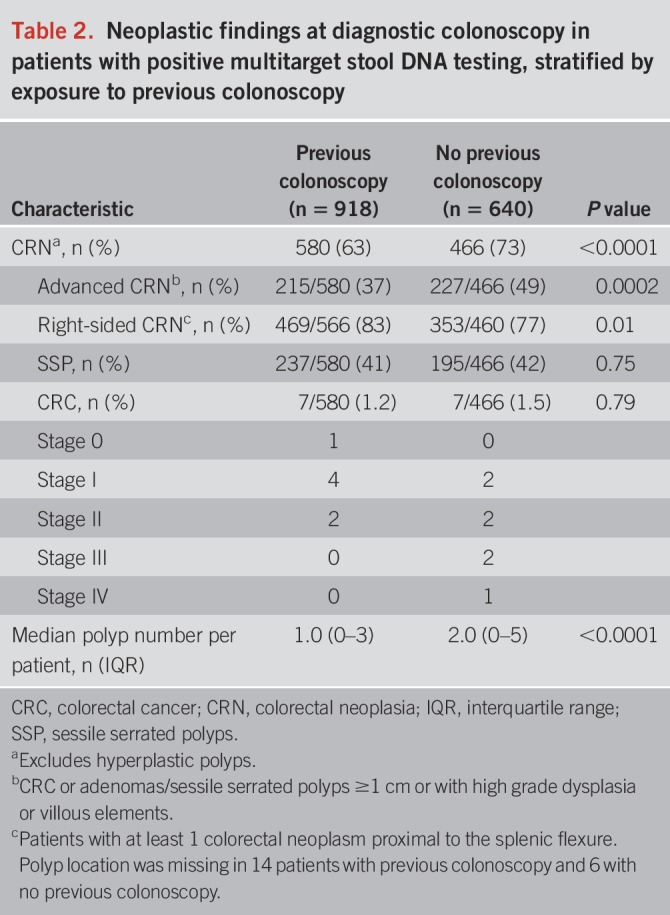
Neoplastic findings at diagnostic colonoscopy in patients with positive multitarget stool DNA testing, stratified by exposure to previous colonoscopy

### Effect of previous colonoscopy on positive predictive value

Of the 849 patients who had undergone previous colonoscopy with known date, the median time since last colonoscopy was 10.3 (IQR 9.5–11.4) years. The finding of any CRN was more common in those without previous colonoscopy (73% [466/640]) than in those with previous colonoscopy (63% [580/918]; *P* < 0.0001) (Table 2). These findings remained significant with and without adjusting for age between the 2 groups (*P* ≤ 0.0001) and when restricting analysis to only those with adequate preparation and complete examination (82% [1,279/1,558]) vs those without (*P* = 0.0089). Significantly more polyps per patient were also detected in the no previous colonoscopy group (median 2.0 [IQR 0–5]) compared with the previous colonoscopy group (median 1.0 [IQR 0–3]; *P* < 0.0001).

Among those with neoplastic lesions, patients without previous colonoscopy had more advanced lesions detected at diagnostic colonoscopy (49% [227/466]) than previously screened individuals (37% [215/580]; *P* = 0.0002). There was no difference between the rates of SSPs between the groups (42% [195/466] vs 41% [237/580] for patients without and with previous colonoscopy, respectively; *P* = 0.79). The proportion of right-sided CRN detected by MT-sDNA was higher in those with previous colonoscopy (83% [469/566]) compared with those without previous colonoscopy (77% [353/460]; *P* = 0.02). Among 381 patients who were older than 60 years and had no previous colonoscopy, MT-sDNA brought advanced neoplasia to clinical attention in 134 (35%) of these previously unscreened patients.

Seven (50%) of the patients with CRC had undergone previous screening colonoscopy at a median interval of 10 (range 6–19) years before. Six (86%) of these patients had right-sided CRC, and all were found to have AJCC stage 0–II stage disease. All but one patient with available previous colonoscopy records (n = 5) had a complete examination with adequate preparation, and none had any documented adenomatous polyps. The remaining patient had an incomplete examination because of an angulated sigmoid colon 19 years earlier without an interim follow-up. In the 7 patients with CRC without previous colonoscopy, 3 (43%) had right-sided CRCs, 4 (57%) had AJCC stage I–II disease, and 3 (43%) had stage III–IV CRC.

## DISCUSSION

In this large, real-world, retrospective cohort study of patients at average risk for CRC, MT-sDNA screening returned positive in 14% of patients who completed the test. The majority (87%) of patients with positive MT-sDNA results underwent diagnostic colonoscopy after testing, with high PPV (67%) of MT-sDNA for any CRN regardless of exposure to previous screening colonoscopy. Among those with CRN detected at diagnostic colonoscopy, the majority had at least one right-sided lesion.

In this study, 41% of patients with positive MT-sDNA testing had not undergone previous CRC screening by colonoscopy, a number that is consistent with previous reports (46,47). Although many of the patients new to CRC screening in our study were first time participants at age 50, the majority (60%) were >60 year old. Put another way, 24% (381/1,558) of patients overall with positive MT-sDNA and a diagnostic colonoscopy were greater than 10 years overdue for screening colonoscopy, suggesting the availability of MT-sDNA may be a catalyst for beginning participation in a CRC screening program.

The overall PPV of MT-sDNA for any CRN in our cohort was 67%, almost 3 times higher than the benchmark adenoma detection rate for average risk screening colonoscopies (48), and 12 percentage points higher than the overall yield of 55% for any CRN reported by Imperiale et al. (41). Overall, the high PPV in our study is likely a result not only of enrichment for neoplasia in the study population created by screening with MT-sDNA but also of the fact that the endoscopist's knowledge of a positive MT-sDNA test has been shown to increase the quality, and therefore the yield of diagnostic colonoscopy (44,49). Because the endoscopists in our study were aware of positive MT-sDNA test results, the higher rates of CRN seen in our study compared with the previous studies of MT-sDNA outcomes (wherein endoscopists were blinded to stool assay results) are likely more reflective of real-world test performance.

Although our study confirmed that the PPV of MT-sDNA for CRN was higher in patients who had not been exposed to previous CRC screening by colonoscopy, the yield remained high in those with previous screening (73% vs 63%). A similar pattern was shown for both advanced CRN and median number of polyps per patient, suggesting that previous endoscopic screening does not appreciably diminish the probability of subsequent detection of neoplastic lesions. Given this finding, the use of MT-sDNA could be a logical complement to screening colonoscopy, whether applied as an interval test or as a method to augment diagnostic yield at colonoscopy, with the goal of interval cancer reduction. Further studies are needed to further evaluate these potential applications.

Importantly, 79% of patients with neoplastic lesions detected by MT-sDNA in our study were found to have at least one right-sided lesion, with proximal neoplastic lesions more prevalent in patients who had undergone previous colonoscopy compared with those who had not (83% vs 77%, *P* = 0.02). The reason for the high prevalence of right-sided neoplasia is likely related to the well-established poorer performance of other CRC screening techniques in the proximal colon, where lesions are inconspicuously flat and less likely to bleed and where bowel preparation is often worse (9–14). This is a limitation that MT-sDNA testing does not face because neoplastic exfoliation has been shown to be similar throughout the colon (50), offering a potential performance advantage over other screening modalities for CRC.

Approximately, 11% of patients in our cohort underwent MT-sDNA testing despite increased risk for CRC based on various factors (Supplemental Table 1, http://links.lww.com/AJG/B401). It was not possible to quantify the reason MT-sDNA was ordered in these patients because of limited documentation of providers' clinical decision-making process in the medical record. However, common reasons included a perception of unfavorable patient risk profile for more invasive methods such as colonoscopy, as well as a possible lack of awareness of CRC risk factors and misunderstanding of the FDA-approved indications for MT-sDNA testing. These findings indicate a need for widespread education of providers regarding appropriate utilization of MT-sDNA.

An important limitation of this study is its retrospective design. Because test-negative patients do not undergo colonoscopy in clinical practice, only PPVs for each endpoint could be estimated, without the ability to directly assess other performance characteristics such as sensitivity, specificity, or false positive rate. However, using the Bayes theorem, one can infer the programmatic screening probability of having a positive MT-sDNA test with no CRN to be roughly 4.7% (14.1% × [100%–67%]); accordingly, fewer than 5% of all MT-sDNA screened patients would result in a positive MT-sDNA test and a colonoscopy showing no CRN. By similar logic, only 10% (14.1% × [100%–28%]) would result in a positive MT-sDNA test and a colonoscopy free from advanced CRN.

Further limitations include differences in demographics about previous colonoscopy exposure. Those with previous screening were more likely to be white and those without were more often exposed to smoking; these imbalances were numerically small, but both are consistent with previous studies examining the socioeconomic trends in CRC screening adherence (51,52). Age was also statistically significantly imbalanced between groups. However, an analysis of the PPV of MT-sDNA for CRN, advanced CRN, and right-sided CRN adjusting for age (both stratifying by age ≥65 and logistic regression utilizing age as a continuous variable) found no significant difference in the PPV for these categories (Supplemental Table 3, http://links.lww.com/AJG/B403). Finally, patients were included regardless of bowel preparation quality to accurately measure compliance with diagnostic colonoscopy. Because suboptimal preparation conditions may have been present for some patients, reducing visibility for right-sided, flat, or serrated polyps, our results may be underestimating the positive predictive value of MT-sDNA. Overall, despite these limitations these data demonstrate the utility of MT-sDNA to screen-detect patients with CRN, including advanced, sessile, and proximal lesions that are variably detected by other conventional screening methods (24–33).

In summary, in this large, real-world study of MT-sDNA screening performance in patients at average risk for CRC, we show that MT-sDNA has a high PPV for CRN at diagnostic colonoscopy, both in patients new to screening and those who have undergone previous colonoscopy. The high prevalence of right-sided CRN detected by MT-sDNA seen in our study suggests potential advantages over other currently available screening modalities for CRC, which could ultimately prove valuable in reducing CRC morbidity and mortality.

## CONFLICTS OF INTEREST

**Guarantor of the article:** John B. Kisiel, MD.

**Specific author contributions:** J.D.E.: data collection, data analysis, drafting and revision of the manuscript, and is the first author. D.W.E.: data collection and data analysis. J.B.: data collection. A.K.: data collection and critical revision. E.R.: data collection. M.E.D.: data collection. K.L.L.: data collection. K.D.: data collection. S.T.: data collection. K.N.B.: data analysis and interpretation. D.W.M.: data analysis and interpretation. D.O.P.: critical revision. M.B.W.: critical revision. S.R.G.: critical revision. L.J.F.: critical revision. P.L.: critical revision. B.B.: critical revision. D.A.A.: critical revision, intellectual content, and funding. J.B.K.: concept and design, data analysis, critical revision, and is the guarantor of the article.

**Financial support:** NIH (CA214679) to J.B.K., Schulze Family Foundation to DAA and J.B.K.

**Potential competing interests:** The Mayo Clinic and Exact Sciences (Madison, WI) own intellectual property under which D.A.A., J.B.K., and D.W.M. are listed as inventors and may receive royalties in accordance with the Mayo Clinic policy. P.L. serves as a chief medical officer for Exact Sciences through a contracted services agreement with the Mayo Clinic. P.L. and the Mayo Clinic have contractual rights to receive royalties through this agreement. B.B. is a consultant to Exact Sciences and had no role in the funding or conduct of the study. J.D.E., D.W.E., J.B., A.K., E.R., D.O.P., M.B.W., S.R.G., & L.J.F. and M.E.D., K.D., K.L.L., S.T., and K.N.B. have no conflicts to disclose.

**Study Highlights**

**WHAT IS KNOWN**

✓ Colonoscopy is the gold standard for CRC screening but has important limitations.
✓ MT-sDNA testing, a noninvasive screening modality for CRC, has high sensitivity and specificity for colorectal neoplasia.
✓ Other CRC screening methods have variable performance in the proximal colon.

**WHAT IS NEW HERE**

✓ Nearly 1 in 4 persons screened by MT-sDNA were more than 10 years overdue to begin participation in colorectal cancer screening.
✓ Adherence to diagnostic colonoscopy after positive MT-sDNA testing is high.
✓ The positive predictive value of MT-sDNA for colorectal neoplasia is high even in those with previous exposure to colonoscopy.
✓ Most patients with polyps detected by MT-sDNA screening have right-sided neoplasia.
